# TIM-3 as a Prognostic Marker and a Potential Immunotherapy Target in Human Malignant Tumors: A Meta-Analysis and Bioinformatics Validation

**DOI:** 10.3389/fonc.2021.579351

**Published:** 2021-02-22

**Authors:** Kui Zang, Liangliang Hui, Min Wang, Ying Huang, Xingxing Zhu, Bin Yao

**Affiliations:** Department of ICU, The Affiliated Huaian No. 1 People’s Hospital of Nanjing Medical University, Huaian, China

**Keywords:** immune checkpoint, TIM-3, tumor, prognosis, immune response

## Abstract

**Background:**

As a novel immune checkpoint molecular, T-cell immunoglobulin mucin 3 (TIM-3) is emerging as a therapeutic target for cancer immunotherapy. However, the predictive role of TIM-3 in cancer remains largely undetermined. This study was designed to investigate the role of TIM-3 in cancer.

**Methods:**

Publications were searched using multiple databases. The hazard ratios (HRs) with 95% confidence intervals (CIs) were calculated. To further confirm the prognostic effect of TIM-3, The Cancer Genome Atlas (TCGA) data were applied. Functional analysis of TIM-3 was also investigated.

**Results:**

28 studies with 7284 patients with malignant tumors were identified. Based on multivariate Cox regression analysis, TIM-3 was an independent prognostic indicator for poor overall survival (OS) (HR= 1.54, 95% CI = 1.19-1.98, *P* = 0.001). However, TIM-3 was not correlated with cancer-specific survival and disease-free survival (DFS). Particularly, TIM-3 showed a worse prognosis in non-small cell lung carcinoma and gastric cancer; but it showed a favorable prognosis in breast cancer. Functional analysis showed that TIM-3 was closely correlated with immune responses such as T-cell activation and natural killer cell-mediated cytotoxicity. Moreover, TIM-3 expression was found to be related to worse OS in 9491 TCGA patients (HR = 1.2, *P* < 0.001), but was not associated with DFS.

**Conclusions:**

TIM-3 was an independent prognostic factor. Meanwhile, TIM-3 played a crucial role in tumor immune responses. This supports TIM-3 as a promising target for cancer immunotherapy.

## Introduction

Cancer is still a global burden issue worldwide. Cancer incidence and mortality are rapidly growing in the world. Based on the GLOBOCAN estimates, an estimated 18.1 million cancer cases were diagnosed, and approximately 9.6 million deaths were due to cancer, in 2018 (1). Until now, general treatment regimens for cancer are used, such as surgery, chemotherapy, radiotherapy, targeted molecular therapy, or immunotherapy. Despite the significant advancements made in cancer treatment, the 5‐year survival rate for cancer patients is not very high (< 70%) (2, 3). Thus, ongoing efforts to identify best practices for cancer treatment and management are needed.

Studies suggest that the development of strategies against biomarkers could be a reasonable and precise therapy approach in tumors (4–8). The immunological aspects are crucial hallmarks for tumor progression and metastasis (9–11). Cancer immunotherapy shows substantial benefits and has become a powerful treatment strategy in controlling many malignant tumors (12, 13). Programmed cell death 1 (PD-1) and programmed cell death ligand 1 (PD-L1) have the potential to become prognostic biomarkers in numerous cancers (14–17). Immune checkpoint therapy, such as PD-1 and cytotoxic T lymphocyte antigen-4 (CTLA-4), has achieved clinical success (18). T-cell immunoglobulin and mucin-dominant containing-3 (TIM-3), also known as hepatitis A virus cellular receptor 2 (HAVCR2), has been reported as an immune-checkpoint molecule (19). TIM-3 plays a vital role in suppressing cytotoxic T lymphocytes (CTL) and Th1 responses and the expression of cytokines such as tumor necrosis factor and interferon-γ (20, 21). TIM-3 regulates innate and adaptive immune responses, possibly exerting either positive or negative effects (22). Numerous studies have reported that TIM-3 is expressed in cancer (23–25). TIM-3 expression is correlated with poor prognosis in many cancers, such as oral squamous cell carcinoma (26), ovarian cancer (27), and gastric cancer (28). However, the role of TIM-3 in clinical cancer studies is still conflicting. For example, Duan 2018 et al. reported no correlation between TIM-3 expression and overall survival (OS) in esophageal squamous cell carcinoma (29). However, Hong 2019 et al. reported a significant association between TIM-3 expression and worse OS in esophageal squamous cell carcinoma (30). Thus, it is of great importance to investigate the prognostic impact of TIM-3 in malignant tumors.

The previous meta-analysis involving only seven studies with a very small population (n=869 patients) evaluated the correlation between TIM-3 expression and OS in solid tumors (31). In recent years, numerous studies (23–27, 29, 30, 32–43) were published that examined the prognosis of TIM-3 expression in various tumors. Here, the aim of the present meta-analysis was to analyze the association of TIM-3 expression with cancer survival (n=28 studies with 7284 patients). Additionally, TCGA data were further used to confirm the results of this meta-analysis, and the potential biological functions of TIM-3 were also investigated. This study will provide more evidence to suggest whether TIM-3 could be a promising target for immunotherapy.

## Materials and Methods

### Literature Search

The PubMed, EMBASE, and Web of Science databases were systematically searched to identify eligible publications before January 29, 2020. The following key words and search terms were applied: “T-cell immunoglobulin and mucin domain containing 3 OR TIM-3 OR TIM3 OR T-cell Ig and mucin domain 3 OR HAVCR2 OR hepatitis A virus cellular receptor 2 OR CD366”, “prognosis OR survival OR outcome OR prognostic”. The reference lists of the included publications were also scanned to find additional potential studies. This meta-analysis was carried out based on the Preferred Reporting Items for Systematic reviews and Meta-Analyses (PRISMA) statement (44).

### Study Selection

The eligible studies were included when they met the following selection criteria: 1) the patients were diagnosed with malignant tumors; 2) studies evaluated the expression of TIM-3 from the original articles; 3) studies reported the prognosis of TIM-3 expression using multivariate Cox regression analysis; 4) the prognostic endpoints, such as OS, disease-free survival (DFS), and cancer-specific survival (CSS), were included; and 5) if the information from an eligible study is not completely reported the corresponding author is contactable *via* email as much as possible. Additionally, for the overlapping sample data from multiple publications, only the latest publication or the most complete study was included. We mainly excluded letters, abstracts, reviews or case reports, cell or animal studies, articles lacking sufficient information or using univariate survival analysis, and articles using RNA-resequencing or microarray data from public databases.

### Data Extraction and Study Quality

The following information was extracted from the available publications, including the first author’s surname, time of publication, country, ethnicity, median/mean age, cancer type, tumor stage, antibody and its sources, detection methods, cut-off values, number of patients, expression frequency, and the survival information of multivariate Cox analysis such as CSS, OS, and DFS. The Newcastle–Ottawa Scale (NOS) was used to assess the quality of the included studies for cohort design (45, 46). Three parameters of quality consisted of the selection of participants (0–4), comparability (0–2), and assessment of outcome (0–3), including a total of nine scores. A study with ≥ six scores was defined as high quality; if s study with < 6 scores was found, it was defined as low quality. Any disagreements in the literature selection and data extraction were resolved by consensus from all authors.

### Prognostic Analysis From TCGA Data in GEPIA2

Gene Expression Profiling Interactive Analysis 2 (GEPIA2) database (http://gepia2.cancer-pku.cn/) was used, including numerous tumor samples from The Cancer Genome Atlas (TCGA). In our study, the relationship between TIM-3 expression and prognosis was further validated from TCGA data.

### Functional Analysis of TIM-3

Association between TIM-3 and genes was analyzed using TCGA data in the GEPIA2 database. Correlation coefficients with > 0.25 were applied for TIM-3. Finally, 733 genes were significantly correlated with TIM-3 (**Table S1**). The GO (Gene Ontology) analysis and KEGG (Kyoto Encyclopedia of Genes and Genomes) pathways were performed to investigate the potential biological functions of the TIM-3 gene using clusterProfiler package (R software, version 3.6.1).

### Statistical Analysis

Data of this meta-analysis were obtained from the original publications. The combined hazard ratios (HRs) with 95% confidence intervals (CIs) were conducted to estimate the association between TIM-3 expression and the prognosis based on multivariate Cox analysis. The heterogeneity assumption was detected using a Cochran’s Q statistic (47). The random-effects model was applied to pool data in this meta-analysis. Substantial heterogeneity was detected when a Q test (*P*-value) was less than 0.1. When significant heterogeneity was found, subgroup analyses were conducted according to age, ethnicity, detection method, tumor stage, tumor type, and sample sizes. A sensitivity analysis was also performed to evaluate the change of heterogeneity and stability by removing an individual study or multiple studies. Egger’s test was applied to assess the possible publication bias (48). Meta-analysis was conducted using Stata software (version 12.0, Stata Corporation, College Station, TX, US).

## Results

### Study Characteristics

**Figure 1:**
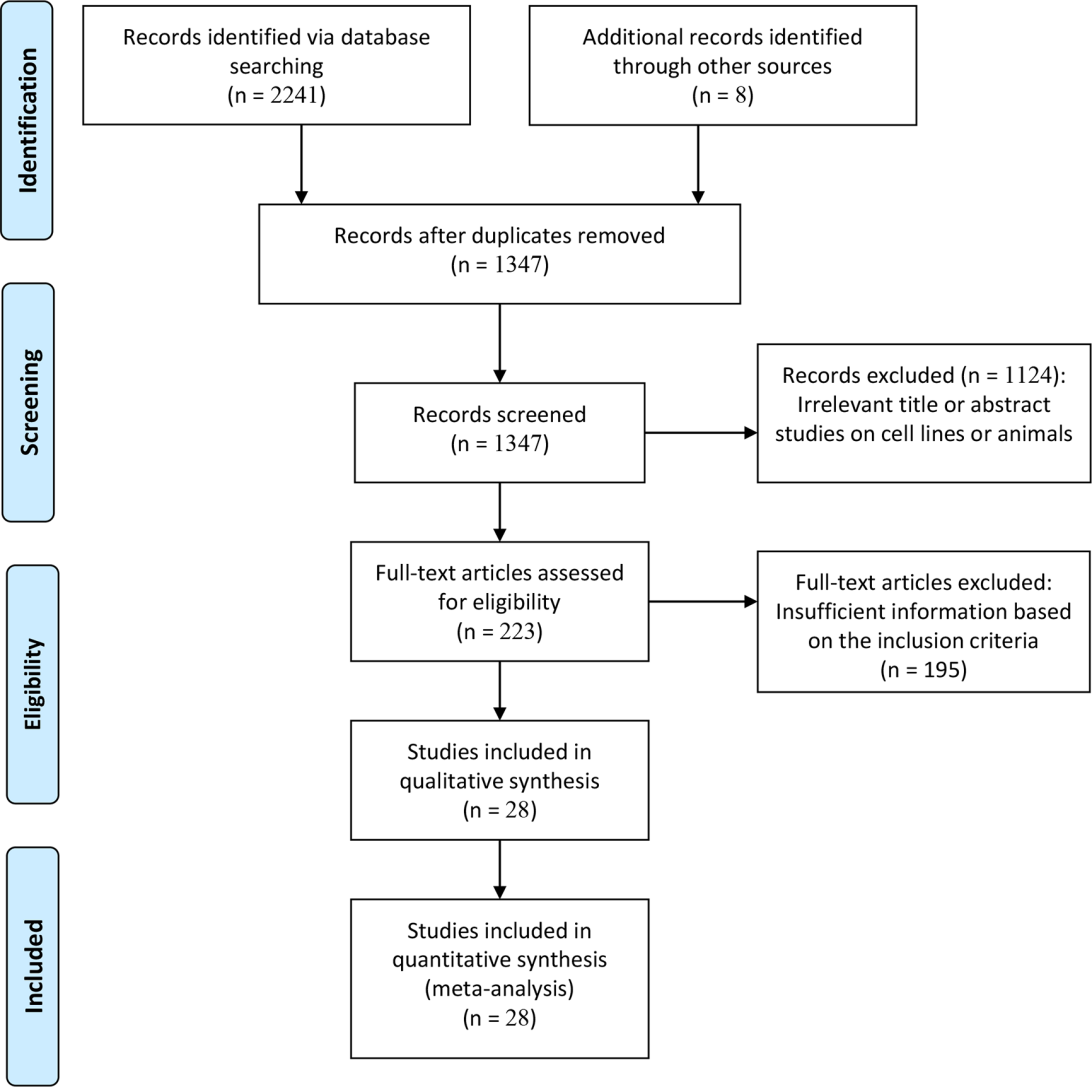
Flowchart diagram of selected articles included in this meta-analysis.

The flow diagram for the details of the study selection is presented in **Figure 1**. According to the inclusion criteria, inappropriate publications were excluded. Finally, 28 studies from 27 publications (23–30, 32–43, 49–55) were identified based on the multivariate Cox analysis, including 7284 patients with malignant tumors. These studies were published between 2012 and 2019. Eligible studies were conducted in China, Korea, Belgium, Canada, USA, and the Czech Republic. Various types of tumors were diagnosed, such as esophageal carcinoma, gastric cancer, sarcoma, non-small cell lung cancer, renal cell carcinoma, triple-negative breast cancer, and prostate cancer. Of these eligible studies, only three articles reported that TIM-3 expression was correlated with worse OS in cervical cancer (n= 43 cases) (54), gastric cancer (n=305 cases) (28), and colorectal cancer (n=201 cases) (50). The remaining 25 studies reported the available HR and CIs, including CSS (n = 2 studies with 3281 cases), OS (n = 22 studies with 3317 cases), and DFS (n = 7 studies with 1240 cases). All studies were considered as high quality based on NOS, with an average score of 7.9 (range, 6-9 scores). The details of the eligible publications are summarized in **Table S2**.

### Prognostic Role of TIM-3 in Cancer

**Figure 2:**
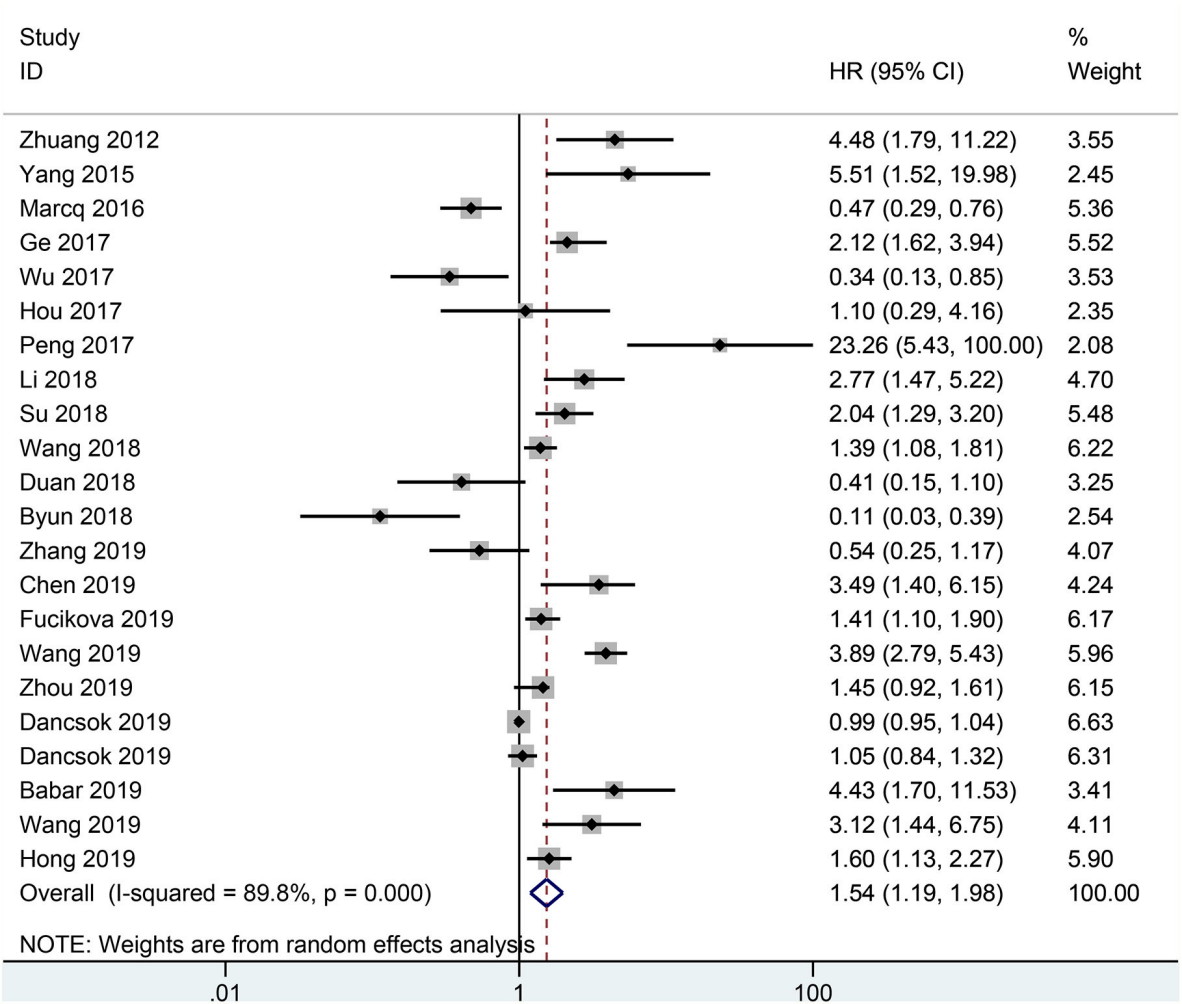
Forest plot of HR with 95% CI for correlation between TIM-3 expression and OS. HR, hazard ratios; CI, confidence interval; OS, overall survival.

**Figure 3:**
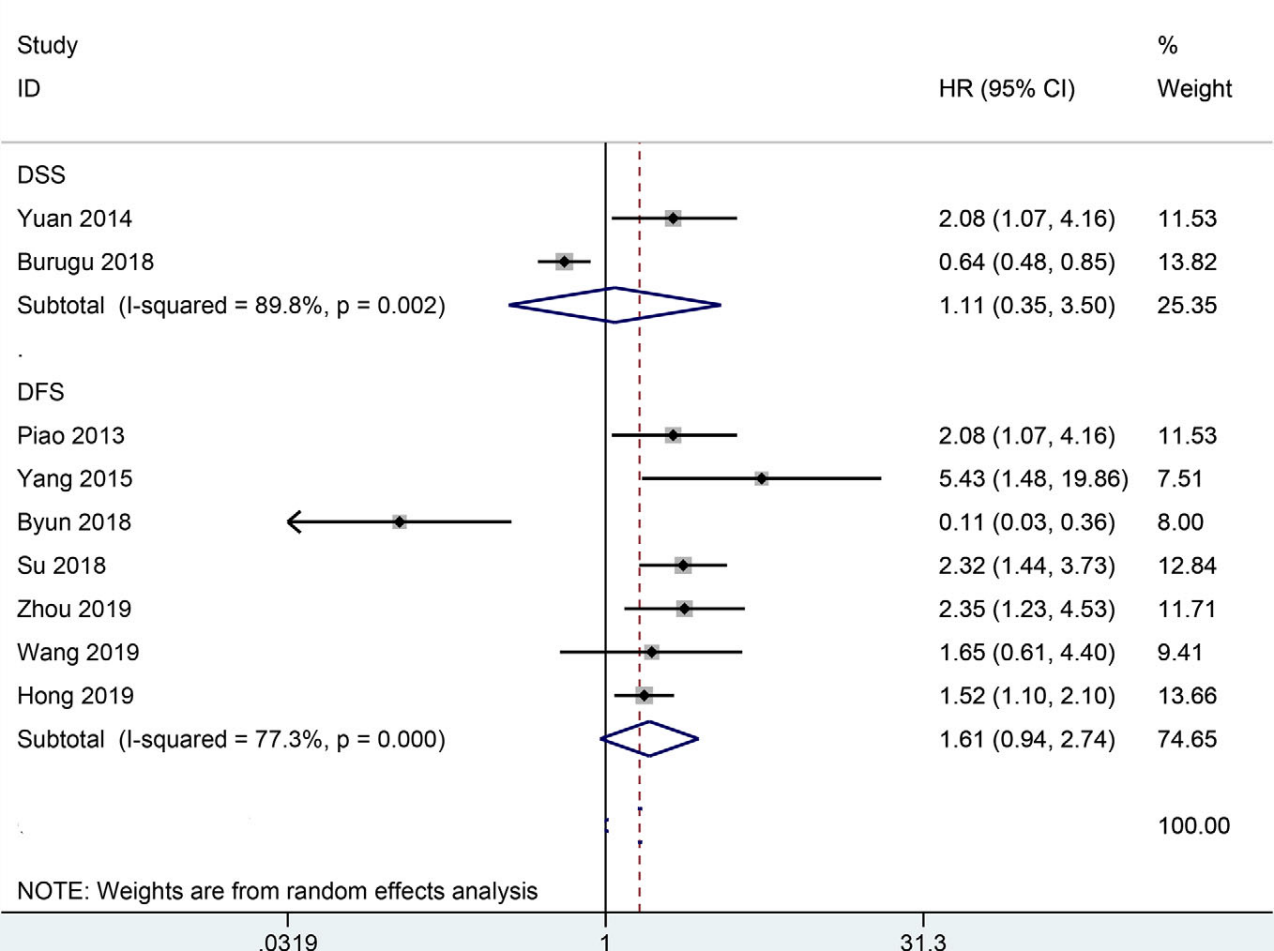
Forest plot of HR with 95% CI for correlation between TIM-3 expression and CSS as well as DFS. HR, hazard ratios; CI, confidence interval; CSS, cancer-specific survival; DFS, disease-free survival.

The result from 22 studies indicated that the expression of TIM-3 led to poorer OS (HR= 1.54, 95% CI = 1.19-1.98, *P* = 0.001) (**Figure 2**), including 3317 malignant tumor patients. Additionally, no significant correlation was found between TIM-3 expression and CSS (n = 2 studies with 3281 patients: HR = 1.11, 95% CI = 0.35-3.50, *P* = 0.863) and DFS (n = 7 studies with 1240 patients: HR = 1.61, 95% CI = 0.94-2.74, *P* = 0.081) (**Figure 3**).

### Subgroup Analyses

Subgroup analyses of OS were further carried out according to multiple potential factors (age, ethnicity, detection method, tumor stage tumor type, and sample sizes), which are summarized in **Table 1**. The results by age group showed that TIM-3 expression was correlated with worse OS in the elder age group (> 60 years: n = 8 studies with 1600 cases: HR = 2.10, 95% CI = 1.34-3.31, *P* = 0.001) and the younger group (≤ 60 years: n = 10 studies with 1348 cases: HR = 1.45, 95% CI = 1.01-2.08, *P* = 0.046). The results grouped by ethnicity showed that TIM-3 expression was associated with poor OS in Asian populations (n = 16 studies with 2452 cases: HR = 1.64, 95% CI = 1.14-2.35, *P* = 0.007), but not in European populations (n = 6 studies with 865 cases: *P* = 0.203). When stratified by detection method, immunohistochemistry (IHC) (n = 17 studies with 2832 cases: HR = 1.34, *P* = 0.05) and non-IHC (n = 5 studies with 485 cases: HR = 2.28, *P* < 0.001) were significant for survival, moreover, three articles with 549 cases reported that TIM-3 expression using IHC was correlated with worse OS (28, 50, 54), suggesting that these two methods showed a relationship with worse OS. By tumor type, TIM-3 expression was not associated with OS in esophageal carcinoma (n = 4 studies with 585 cases: *P* = 0.449), sarcoma (n = 3 studies with 780 cases: *P* = 0.232), and renal cell carcinoma (n = 2 studies with 345 cases: *P* = 0.769), but was related to favorable OS in triple-negative breast cancer (n = one study with 109 cases: HR = 0.1129, *P* = 0.0006) and prostate cancer (n = one study with 139 cases: HR = 0.336, *P* = 0.021); TIM-3 was related to worse OS in non-small cell lung cancer (n = 2 studies with 253 cases: HR = 2.72, *P* = 0.008) and gastric cancer (n = one study with 587 cases: HR = 1.395, *P* = 0.012).

**Table 1:** Stratification analysis of TIM-3 for overall survival.

Subgroups	HR (95% CI)	Heterogeneity (*P*)	*P* value	Studies	Patients
**Age (years)**					
≤ 60	1.45 (1.01-2.08)	<0.001	0.046	10	1348
> 60	2.10 (1.34-3.31)	<0.001	0.001	8	1600
Not clear	0.97 (0.50-1.89)	<0.001	0.926	4	369
**Ethnicity**					
Asian	1.64 (1.14-2.35)	<0.001	0.007	16	2452
European	1.23 (0.90-1.68)	<0.001	0.203	6	865
**Method**					
IHC	1.34 (1.00-1.79)	<0.001	0.05	17	2832
Not IHC	2.28 (1.53-3.40)	0.03	< 0.001	5	485
**Tumor stage**					
Stage 2-3	1.98 (1.30-3.03)	0.36	0.001	2	165
Stage 1-3	1.01 (0.24-4.27)	<0.001	0.994	3	514
Stage 1-4	2.06 (1.19-3.57)	<0.001	0.01	9	1384
NA	1.19 (0.91-1.55)	<0.001	0.209	8	1254
**Tumor type**					
Esophageal cancer	1.38 (0.60-3.20)	0.008	0.449	4	585
NSCLC	2.72 (1.29-5.72)	0.132	0.008	2	253
Sarcoma	1.20 (0.89-1.61)	0.004	0.232	3	780
Renal cell carcinoma	1.29 (0.23-7.28)	0.002	0.769	2	345
Triple-negative breast cancer	0.1129 (0.0323–0.3948)	NA	0.0006	1	109
Gastric cancer	1.395 (1.078−1.807)	NA	0.012	1	587
Others	1.98 (1.14-3.44)	<0.001	0.015	9	658
**Sample sizes**					
>100	1.18 (0.90-1.55)	<0.001	0.237	10	2579
≤100	2.21 (1.37-3.55)	<0.001	0.001	12	738

HR, hazard ratio; CI, confidence interval; IHC, immunohistochemistry; NA, no applicable; NSCLC, non-small cell lung cancer.

### Heterogeneity Analysis

According to the available information, we performed subgroup analyses to explore possible sources of heterogeneity. **Table 1** shows the results of subgroup analyses, and we found that all *P* values among each subgroup were not > 0.1 for heterogeneity. Our results suggested that these factors (age, ethnicity, detection method, tumor stage, tumor type, and sample sizes) could not explain the potential heterogeneity sources. We further performed a sensitivity analysis to assess the change of the pooled results and heterogeneity. Studies by Zhuang et al. (55), Marcq et al. (49), Wu et al. (42), Peng et al. (25), Byun et al. (36), Duan et al. (29), Chen et al. (35), Babar et al. (33), Wang et al. (26), Dancsok et al. (23), and Zhang et al. (24) were omitted. The re-calculated result from the remaining studies showed that TIM-3 expression was still significantly correlated with shorter OS (HR= 1.71, 95% CI = 1.44-2.04, *P* < 0.001), with no heterogeneity (*P* = 0.102).

### Publication Bias

A slight publication bias was found between TIM-3 and OS (*P* = 0.025), but no publication bias was detected between TIM-3 and DFS (*P* = 0.75) (**Figure S1**).

### Prognostic Value of TIM-3 From TCGA Data

**Figure 4:**
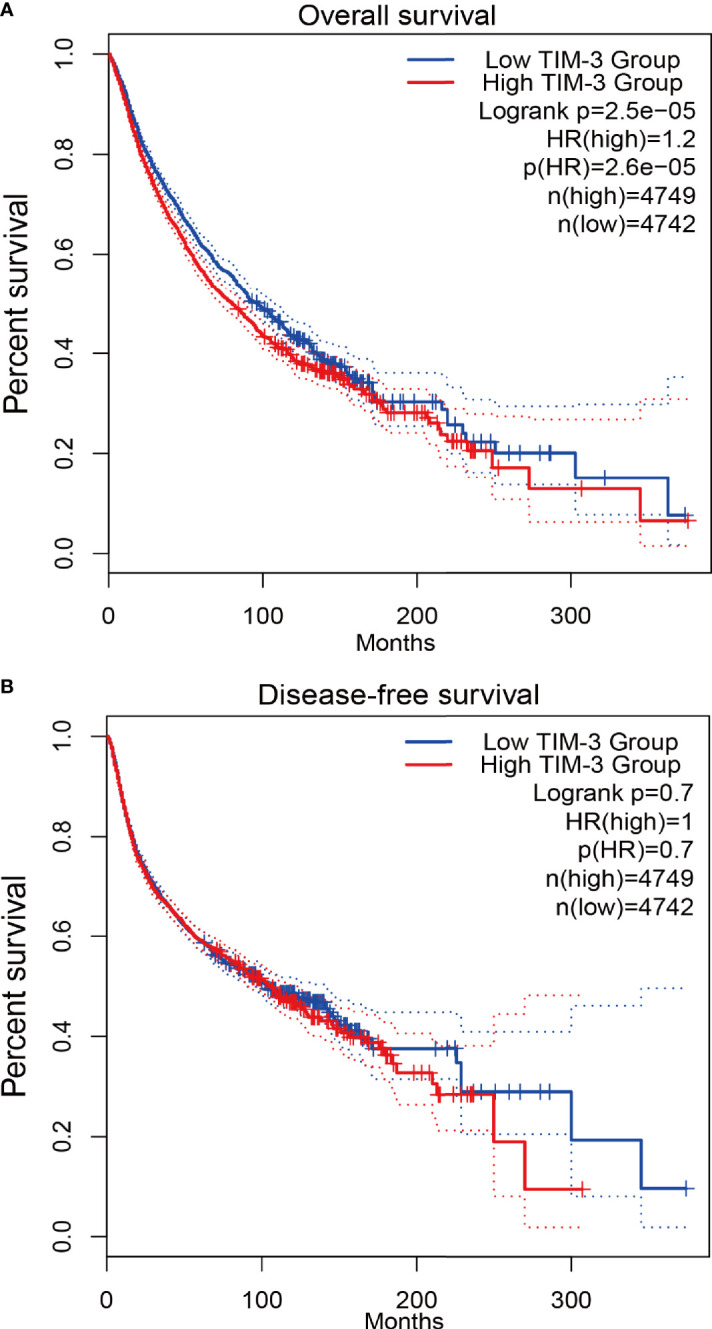
Survival analysis of TIM-3 expression from TCGA validation data in 9491 cancer patients. **(A)** Overall survival (OS). **(B)** Disease-free survival (DFS).

To further confirm the relationship between TIM-3 expression and patients’ prognosis in cancer, we conducted the survival analysis using TCGA data from the GEPIA2 database. The results demonstrated that there was a significant association between TIM-3 expression and worse OS in 9491 cancer patients (HR = 1.2, *P* < 0.001), but no correlation was observed between TIM-3 expression and DFS in 9491 cancer patients (*P* = 0.7) (**Figure 4**).

### Biological Functions of the TIM-3 Gene

**Figure 5:**
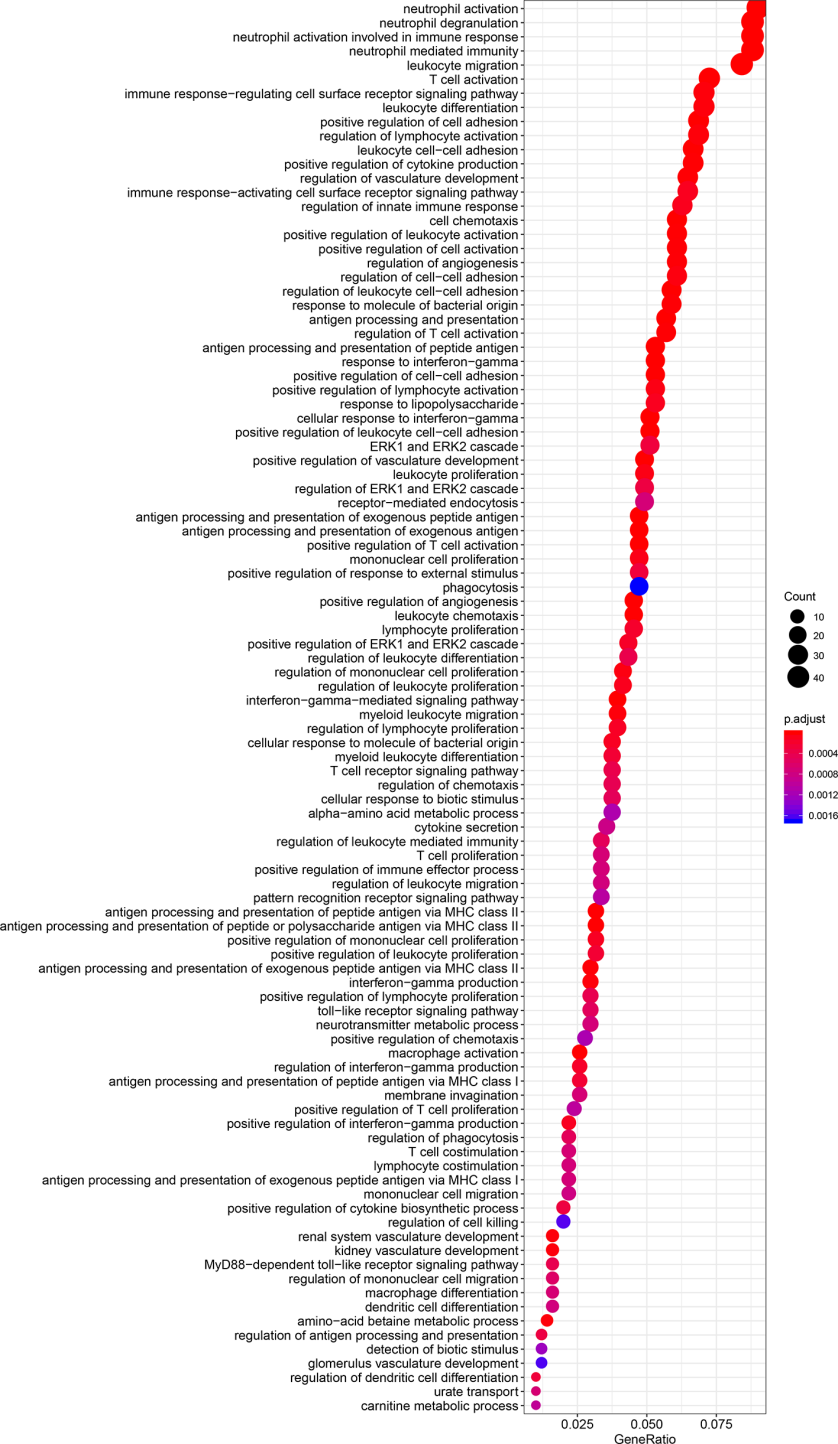
TIM-3 involves in immune regulation from the GO (Gene Ontology) analysis.

**Figure 6:**
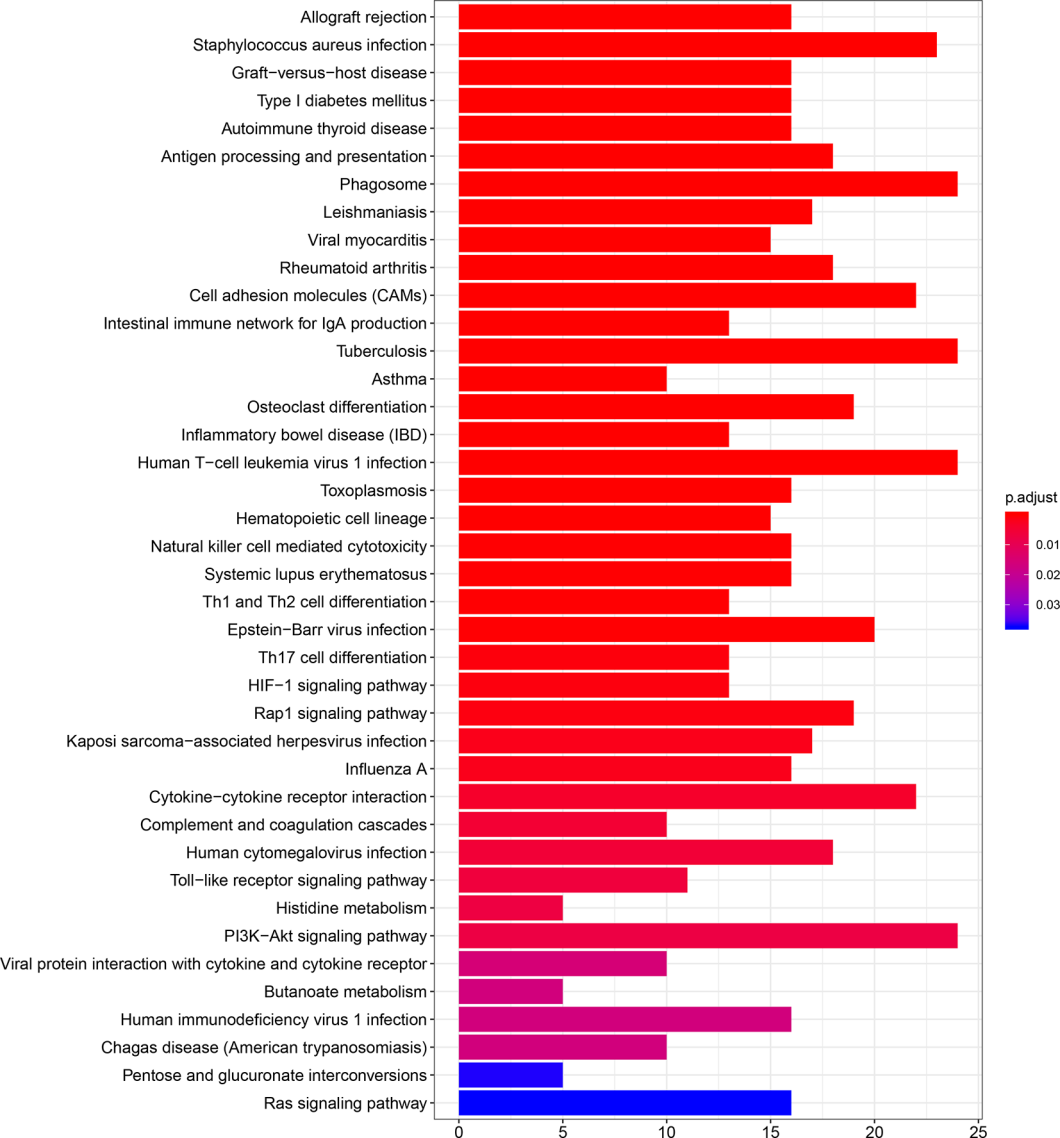
TIM-3 involves in immune regulation from the KEGG (Kyoto Encyclopedia of Genes and Genomes) pathways.

The results of GO and KEGG analyses demonstrated that TIM-3 was related to a wide variety of immune responses, such as T-cell activation, response to interferon-gamma, neutrophil-mediated immunity, macrophage differentiation, regulation of the cell surface receptor signaling pathway, innate immune response and dendritic cell differentiation, antigen processing and presentation of peptide antigen *via* MHC class I/II, toll−like receptor signaling, cytokine-cytokine receptor interaction, Th1 and Th17 cell differentiation, Th2 cell differentiation, and natural killer cell-mediated cytotoxicity (**Figures 5** and **6**). These results suggested that TIM-3 had a vital role in immune regulation in cancer.

## Discussion

Cancer is a major public health problem worldwide, with high incidence and mortality rates (1). The mechanism of tumor development is complicated. This is generally thought to be closely related to the immune system. The immune system has a crucial role in the development, progression, and metastasis of cancer (9–11, 56). Immune checkpoints are inhibitory signals used by the immune system that have a functional role in the regulation of the immune response and maintaining self-tolerance (57, 58). Immune checkpoint blockade (ICB) PD-1 and CTLA-4 have been shown as promising approaches for tumor immunotherapy and are used to treat some cancers (59). TIM-3 is identified as a crucial immune checkpoint molecule and has been demonstrated as a marker of T-cell exhaustion (20, 57). TIM-3 could also shift the immune responses by the regulation of Th1 CD4 T cells and cytotoxic CD8 T cells, which may dampen the anti-tumor immune response through modulating the T-cell activity (57, 60). TIM-3 may play an essential role in the development and progression of cancer (40, 61–63). Studies have reported that the expression of TIM-3 can be frequently detected in malignant tumors (23–25). TIM-3 expression is related to worse prognosis in some cancers, such as gastric cancer (38) and ovarian cancer (27), but is not correlated with prognosis in some cancers, such as renal cell carcinoma (24) and sarcoma (23). Therefore, the role of TIM-3 in malignant tumors is still largely uncertain. In the current work, we determined the expression of TIM-3 on the prognostic impact of patients with malignant tumors.

Fang et al. reported that TIM-3 single nucleotide polymorphisms (SNPs) were correlated with an increased cancer risk (case-control studies with 4852 participants) (64). Zhang et al. reported that TIM-3 expression was associated with shorter OS in solid tumors (n=869 patients) (31). Qin et al. reported that TIM-3 expression was relevant to poor OS in solid tumors (n=3072 patients) (65). Data of the prognostic evaluation from the previous meta-analyses included univariate and multivariate Cox analysis (31, 65). To our knowledge, the present work was a comprehensive meta-analysis of 28 studies with a very large population for the assessment of TIM-3 expression with survival in malignant tumors (n=7284 patients). Moreover, data using multivariate Cox analysis could reduce the effect of confounding factors (4). Thus, our meta-analysis only included the prognostic data based on multivariate analysis. We found that TIM-3 expression was significantly correlated with reduced OS in cancer. However, TIM-3 expression was not correlated with DFS and CSS, which were consistent with the previous publications for OS (25–28, 30, 32, 33, 35, 38–40, 43, 50, 51, 54, 55) and for DFS (32). Moreover, further TCGA data (n=9491 cases) also confirmed a significant relationship between TIM-3 expression and poor OS, and no relationship between TIM-3 expression and DFS in cancer. TIM-3 may have a function in promoting tumor cell proliferation, migration, and invasion (66, 67). TIM-3 facilitates the initiation of tumor and tumor-promoting activities (66, 68). The interactions between TIM-3 and its ligands inhibit Th1 and Th17 responses and NK cell-mediated cytotoxicity, resulting in immune tolerance (68–70). The TIM-3-galectin-9 pathway could be involved in the prevention of anti-tumor immunity (71). TIM-3 expression is correlated with resistance to PD-1 blockade in preclinical models (72). TIM-3 regulates immune responses in cancer (20). We also observed a similar finding; TIM-3 was closely related to immune regulation, such as T-cell activation, response to interferon-gamma, neutrophil-mediated immunity, macrophage differentiation, regulation of the cell surface receptor signaling pathway, innate immune response and dendritic cell differentiation, antigen processing and presentation of peptide antigen *via* MHC class I/II, toll−like receptor signaling, cytokine-cytokine receptor interaction, Th1, Th17 and Th2 cell differentiation, and natural killer cell-mediated cytotoxicity. These analyses suggest that TIM-3 could become an independent prognostic marker for predicting worse OS, and targeting TIM-3 is a potentially effective approach for cancer immunotherapy.

Although no association was found between TIM-3 expression and CSS in our work, the result should be considered with caution. Among these two studies, Yuan 2014 et al. reported that TIM-3 expression was correlated with worse CSS in clear cell renal cell carcinoma (n=137 cases) (52), but Burugu 2018 et al. reported that TIM-3 expression was related to favorable CSS in a large cohort of breast cancer (> 3000 cases) (37). Additionally, we also observed similar findings in other prognostic endpoints. TIM-3 expression was associated with worse OS in clear cell renal cell carcinoma (n= 182 cases) by Wang et al. (32). TIM-3 expression was correlated with better OS and DFS in triple-negative breast cancer (n= 109 cases) by Byun et al. (36). These results showed that TIM-3 might be an independent positive prognostic factor in breast cancer and be an independent negative prognostic factor in clear cell renal cell carcinoma. Further relevant studies are necessary to confirm these findings in the future.

Stratification by age showed that TIM-3 was significantly correlated with poor OS in the elder group (*P* = 0.001). A slight association between TIM-3 and OS was observed in the younger group (*P* = 0.046). Some studies demonstrate an age effect going in different directions (73–75). For example, Zhang et al. reported that age (the elder group) was associated with a poorer prognosis (73). In the future, additional studies with larger sample sizes are essential to further confirm whether TIM-3 is closely correlated with worse prognosis in younger patients. Subgroup analysis by ethnicity demonstrated that TIM-3 expression was related to worse OS in Asian populations, but not in European populations. When stratified by tumor type, TIM-3 expression was not associated with OS in esophageal carcinoma, sarcoma, and renal cell carcinoma, but was correlated with favorable OS in triple-negative breast cancer; TIM-3 was related to worse OS in non-small cell lung cancer and gastric cancer. Evidence from some previous publications is consistent with the present results, such as esophageal carcinoma (no association) (29, 41), sarcoma (no association) (23), renal cell carcinoma (no association) (24), non-small cell lung cancer (worse OS) (39, 55), gastric cancer (worse OS) (28, 38), and breast cancer (better CSS) (37). We found the correlation between TIM-3 expression and worse prognosis among most cancers, such as lung, gastric, cervical, ovarian, colorectal, bladder, pancreatic, hepatocellular, and oral squamous cell carcinomas, as well as diffuse large B-cell lymphoma. Moreover, there was an association between TIM-3 expression and favorable prognosis among several cancers, such as malignant pleural mesothelioma and breast cancer, suggesting that TIM-3 may be either a negative prognostic factor or a positive prognostic factor based on different tumor types. Studies show TIM-3 regulates immune responses, possibly exerting either positive or negative effects (22). Thus, more studies are necessary among different tumor types in the future.

The current study had some limitations. First, most studies (n=22) used Asian participants; the remaining six studies used Europeans. Other ethnic groups, such as Africans, were inadequately represented. Second, slight publication bias was measured between TIM-3 and OS (*P* = 0.025), possibly because three publications only reported positive results, but did not provide available HR values with 95% CI. Thus, the pooled HR was not included in these three publications. Third, although subgroup analyses and sensitivity analyses were performed to explore the potential sources of heterogeneity, factors such as age, ethnicity, detection method, tumor stage, tumor type, and sample sizes failed to explain the possible heterogeneity sources. The detailed reasons for heterogeneity were not very certain. The unavoidable reasons, such as different or unclear cut-off values of TIM-3 expression and different follow-up time points, may cause the potential sources of heterogeneity. Fourthly, only one study used a prospective design (32); more prospective studies are needed.

Although our study had some limitations, this work was still the largest meta-analysis that incorporated 28 studies with over 7000 patients with malignant tumors. Moreover, over 9000 cancer patients from TCGA data were also applied to confirm our results.

The present study provided more evidence that TIM-3 expression was significantly associated with worse OS, and it might be a useful prognosticator in malignant tumors. Biological functions also showed that TIM-3 played a key role in immune regulation. Immune checkpoint TIM-3 could be a valuable immunotherapy target. Future prospective studies are required to validate the results.

## Data Availability Statement

The original contributions presented in the study are included in the article/**Supplementary Material**. Further inquiries can be directed to the corresponding author.

## Author Contributions

LH and KZ provided the study conception and design. KZ and LH contributed to the drafting of the article and final approval of the submitted version. All authors provided the analyses and interpretation of the data and completion of figures and tables. All authors contributed to the article and approved the submitted version.

## Conflict of Interest

The authors declare that the research was conducted in the absence of any commercial or financial relationships that could be construed as a potential conflict of interest.

## Supplementary Material

Supplementary Figure 1 Publication bias by using Egger’s test.
