# Supplementary figures and images for: TIM-3 as a Prognostic Marker and a Potential Immunotherapy Target in Human Malignant Tumors: A Meta-Analysis and Bioinformatics Validation
Source: Front Oncol. 2021 Feb 22;11:579351. doi: 10.3389/fonc.2021.579351 (PMC7938756; doi:10.3389/fonc.2021.579351)

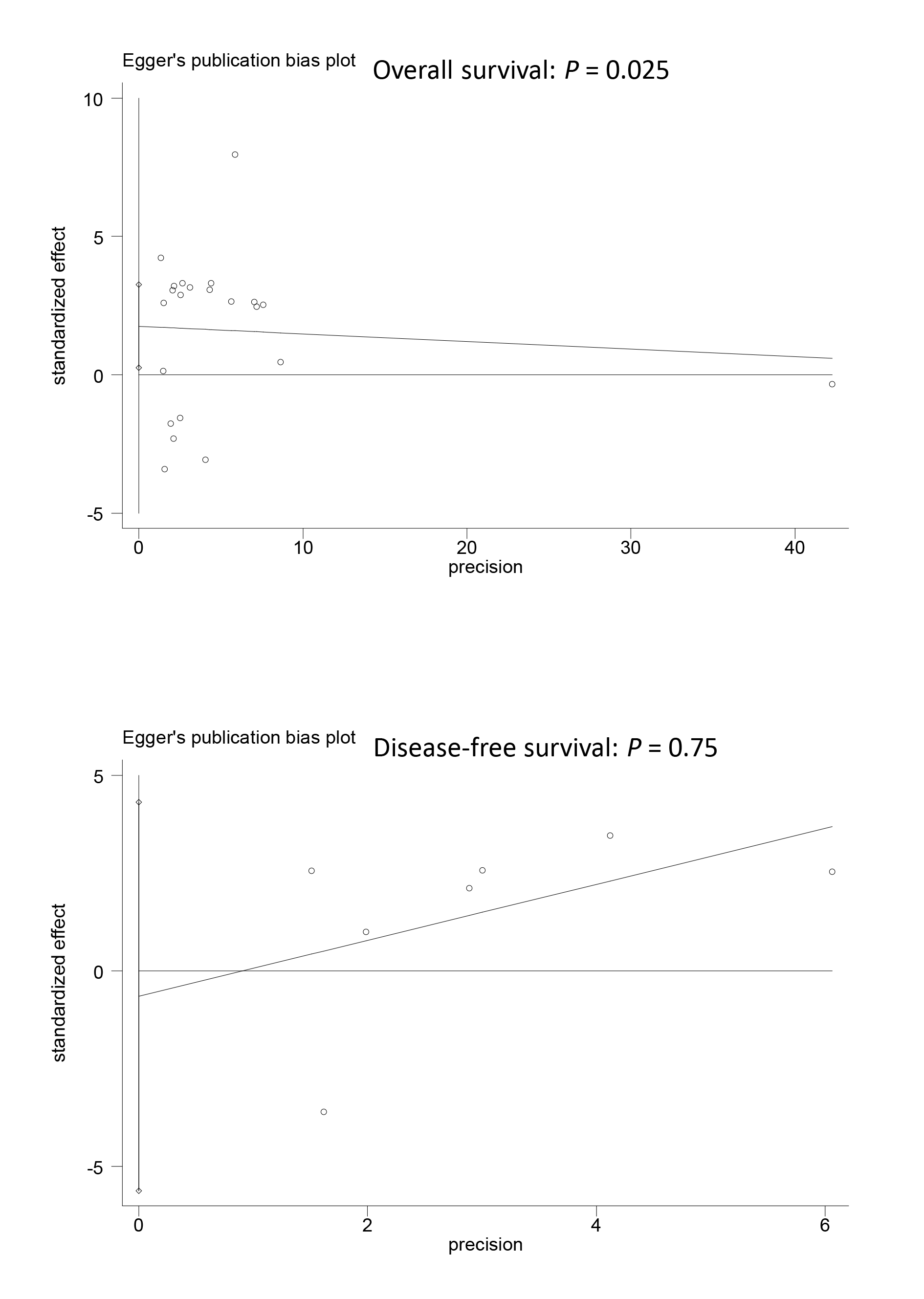

Supplement: Supplementary Figure 1 — Publication bias by using Egger’s test. [file Image_1.tif]
