# Supplementary material for: TIM-3 as a Prognostic Marker and a Potential Immunotherapy Target in Human Malignant Tumors: A Meta-Analysis and Bioinformatics Validation
Source: Front Oncol. 2021 Feb 22;11:579351. doi: 10.3389/fonc.2021.579351 (PMC7938756; doi:10.3389/fonc.2021.579351)
Supplement: Supplementary file 2 [file Table_1.docx]

Table S1. The selected genes with a significant association of TIM-3.

| **Gene symbol** |
| --- |
| DAB2 |
| TCN2 |
| SLC22A2 |
| GIMAP4 |
| CNDP2 |
| ADAP2 |
| GPR65 |
| TLR3 |
| ACAD11 |
| SLC28A1 |
| HAVCR1 |
| FCGR3A |
| MS4A6A |
| ABI3 |
| MSR1 |
| C3AR1 |
| TMEM140 |
| ARSB |
| ENPEP |
| GIMAP6 |
| SLC3A1 |
| LIPA |
| CD68 |
| GAL3ST1 |
| CUBN |
| SLC5A10 |
| AGXT2 |
| SLC16A4 |
| PLIN2 |
| AC090286.4 |
| SLC7A7 |
| HLA-DPA1 |
| TLR7 |
| FBXL5 |
| SNX29 |
| SLC6A13 |
| VCAM1 |
| SLC5A12 |
| TYROBP |
| HLA-DRA |
| CD86 |
| GLRX |
| C11orf54 |
| RP11-807H17.1 |
| LRRC25 |
| SOD2 |
| CSF1R |
| HLA-DPB1 |
| SLC2A5 |
| TNIP1 |
| RP11-134L10.1 |
| ASPA |
| SMPDL3A |
| TMEM37 |
| RP11-252A24.5 |
| SLC22A5 |
| NAT8 |
| CCR5 |
| CDK18 |
| RP1-60O19.1 |
| BBOX1 |
| LAPTM5 |
| C1QC |
| ENPP3 |
| DGCR5 |
| LRP2 |
| CCR1 |
| MS4A4A |
| GNGT2 |
| GIMAP8 |
| GIMAP1-GIMAP5 |
| CD300A |
| GALNT14 |
| LY86 |
| FCGR1A |
| SLC47A1 |
| SEC14L6 |
| C1QB |
| ADORA3 |
| LINC01507 |
| FBXO17 |
| IL10RA |
| GIMAP1 |
| SNX10 |
| CD74 |
| RP11-296I10.3 |
| FPR3 |
| RP11-736E3.1 |
| RBP5 |
| ST8SIA4 |
| RGL1 |
| NEK6 |
| PILRA |
| SIGLEC7 |
| SLCO4C1 |
| CLEC18B |
| SIGLEC9 |
| ARHGAP24 |
| SEMA5B |
| TM6SF1 |
| PECAM1 |
| HMOX1 |
| ZNF395 |
| FCGR1B |
| BNIP3L |
| AC098614.2 |
| AP001626.1 |
| TINAG |
| FCGR1C |
| TREM2 |
| ALPK2 |
| C2orf15 |
| RP11-255M6.1 |
| HLA-DOA |
| C1orf162 |
| LILRB4 |
| TFEC |
| NOX4 |
| C9orf66 |
| ZNF826P |
| COL23A1 |
| RNASE6 |
| FMO1 |
| SLC15A4 |
| SIRPAP1 |
| RN7SL138P |
| SLC16A12 |
| RNF130 |
| P2RY13 |
| SLC37A4 |
| TMEM27 |
| BTN3A2 |
| TMEM200A |
| ABCA1 |
| SLC17A3 |
| GPR34 |
| LEPROTL1 |
| CLEC18C |
| CD4 |
| AVPR1B |
| GIMAP7 |
| DMGDH |
| CRYZ |
| TMCC1 |
| RP11-848P1.3 |
| IGSF6 |
| FUT11 |
| TM4SF18 |
| CLEC18A |
| SKAP2 |
| GATM-AS1 |
| HLA-E |
| CYP2J2 |
| ITGB2 |
| NRP1 |
| SULT1C4 |
| NPY6R |
| FPR1 |
| RP11-505K9.4 |
| VSIG4 |
| BTN3A1 |
| RHOBTB1 |
| NAT8B |
| SLC22A11 |
| ANXA4 |
| SIGLEC10 |
| TLR1 |
| GIMAP2 |
| RP11-283I3.4 |
| C5orf15 |
| HLA-DRB1 |
| LINC01320 |
| RP11-756G20.1 |
| SLC22A4 |
| KSR1 |
| RP11-115N4.1 |
| FAM149A |
| SPI1 |
| RCBTB2 |
| CTC-251I16.1 |
| SLC17A1 |
| FLT1 |
| KRBA1 |
| EGLN3 |
| TNFSF12 |
| CYBB |
| BTN3A3 |
| CLIC4 |
| LINC00887 |
| CD53 |
| CDC42SE2 |
| ANGPTL4 |
| MSRA |
| BHMT2 |
| TAL2 |
| SLC6A3 |
| PDZK1 |
| AIF1 |
| TNFAIP6 |
| STARD8 |
| CLCN5 |
| CTC-360G5.9 |
| EPB41L4A |
| HLA-DMA |
| SLC7A9 |
| TNFRSF1B |
| TNFAIP8L2 |
| CTD-2651B20.4 |
| NR3C1 |
| CDH6 |
| BCO1 |
| PKD2 |
| RP4-764O22.1 |
| ABC12-49244600F4.4 |
| DDO |
| GIMAP5 |
| C7orf49 |
| PLEKHA2 |
| RP11-133F8.2 |
| SLCO2B1 |
| KBTBD11 |
| GBA3 |
| NLRC4 |
| MGAM |
| C1QA |
| SIGLEC8 |
| RP5-856G1.1 |
| B2M |
| LINC01428 |
| TSPAN12 |
| CMBL |
| KL |
| IGFBP7 |
| ADPRH |
| ZNF366 |
| HMGN2P47 |
| ANO6 |
| RP11-492D6.3 |
| JAK1 |
| LST1 |
| CD163 |
| CD70 |
| CD93 |
| GLS |
| ABLIM3 |
| SIRPA |
| SLA |
| BLOC1S5 |
| RP11-205M3.3 |
| FAM134B |
| RP11-478K15.6 |
| HRH2 |
| PTH2R |
| MAF |
| CRTAM |
| ARRB2 |
| HLA-DQA1 |
| RB1 |
| RP11-13J10.1 |
| SAMSN1 |
| NPR3 |
| ANKRD33B |
| GLB1L |
| RAB11FIP5 |
| FCGR2A |
| SPATA18 |
| LILRB1 |
| MLYCD |
| UGT2A3 |
| KANSL1L |
| TLR4 |
| APBB1IP |
| RP4-560B9.4 |
| FCER1G |
| EGOT |
| TRABD2B |
| RP1-261G23.7 |
| IL18BP |
| HSD3B7 |
| RP11-723G8.1 |
| RAB8B |
| DYSF |
| PFKP |
| GZMK |
| IL12RB1 |
| CTSO |
| HIPK2 |
| RP11-389C8.2 |
| FAM26F |
| DOCK4 |
| ITM2B |
| ADGRL4 |
| TRIM22 |
| NPL |
| ADAMTS9-AS1 |
| FZD4 |
| AC069363.1 |
| HPCAL1 |
| RP11-407N17.3 |
| STK32B |
| UGT2B26P |
| RAB11FIP3 |
| CECR1 |
| SIGLEC1 |
| HLA-DRB6 |
| KIAA1191 |
| RP13-452N2.1 |
| SMIM2-AS1 |
| LCP2 |
| CRIM1 |
| CLEC4A |
| ANKRD13A |
| RASSF4 |
| VEGFA |
| AP000445.1 |
| ESM1 |
| HCLS1 |
| PRKAA2 |
| SLAMF8 |
| AC003984.1 |
| ACMSD |
| RP11-70D24.3 |
| SH3BP2 |
| RAB29 |
| RP11-736K20.5 |
| PLA2G15 |
| HILPDA |
| FTH1P22 |
| POU3F3 |
| LGALS2 |
| CTSS |
| BIVM |
| ARL2BP |
| ACSM2A |
| GIPC2 |
| TSPAN33 |
| HOXA4 |
| SH2B3 |
| DARS |
| UGT1A9 |
| WWC2-AS2 |
| MGLL |
| WIPF1 |
| EVI2A |
| TMEM174 |
| TBXAS1 |
| AF127936.5 |
| TMEM176A |
| LEPROT |
| ACAA2 |
| FASLG |
| SELPLG |
| RP11-798K3.2 |
| C5AR1 |
| GPR4 |
| TTC21B-AS1 |
| L3MBTL4-AS1 |
| MIOX |
| AP002954.4 |
| TLN2 |
| RP11-64B16.4 |
| VWF |
| NR1H4 |
| FMNL3 |
| PDZK1IP1 |
| FGL2 |
| SPG20 |
| KDR |
| AC011899.9 |
| SLC22A13 |
| IFI30 |
| HLA-B |
| IKBIP |
| MTRR |
| PICALM |
| AC091199.1 |
| BICC1 |
| AL035610.1 |
| RP11-235C23.6 |
| UBA5 |
| NRIP2 |
| USH1C |
| TRIM38 |
| TMEM106A |
| TGFBR2 |
| SEC14L1 |
| NR2F2-AS1 |
| CSF2RA |
| HNMT |
| NCEH1 |
| TMED5 |
| ARHGAP25 |
| MAP3K14 |
| RGS1 |
| PSAP |
| TMEM176B |
| DRAM1 |
| RP11-14C10.5 |
| RP11-474B16.1 |
| GPR82 |
| GIT2 |
| AD000671.6 |
| TNS1 |
| ETS1 |
| SLC35F6 |
| FAHD1 |
| LAP3 |
| RP11-736K20.4 |
| SLC16A12-AS1 |
| RP11-598F7.6 |
| LRRC37A7P |
| TPP1 |
| PFKFB3 |
| AC107218.3 |
| ADAMTS9-AS2 |
| PPP2R3A |
| SLC6A12 |
| HLA-DQA2 |
| CARD8-AS1 |
| ALOX5AP |
| RP11-64B16.2 |
| DOCK8 |
| PIK3AP1 |
| ARHGAP42 |
| CMKLR1 |
| RP11-672A2.4 |
| S1PR1 |
| RP11-386I14.2 |
| SYNPO |
| PRF1 |
| ENG |
| GAS2L3 |
| STOM |
| AGPAT3 |
| HNF1B |
| FYB |
| NIPA2P1 |
| MAT2B |
| CYTH4 |
| RP11-91K11.2 |
| NDUFA4L2 |
| RP11-19P22.8 |
| MAPRE2 |
| NECAP2 |
| SCIMP |
| INSIG2 |
| RP11-155G14.6 |
| PBLD |
| ANGPT2 |
| PCDH12 |
| GUCY1B3 |
| TNS3 |
| AP001056.1 |
| RP11-1072A3.3 |
| FCGR2B |
| ADORA3 |
| LDB2 |
| CASS4 |
| AGBL3 |
| BBS12 |
| CD200R1 |
| CALCRL |
| CD300C |
| VASH1 |
| CTD-2034I21.1 |
| AP001055.6 |
| RP11-1151B14.4 |
| APLN |
| CNPPD1 |
| CYP4V2 |
| EHHADH |
| RNF13 |
| SAMD3 |
| FLVCR2 |
| ECSCR |
| DOK2 |
| TICAM2 |
| ACADM |
| ACSM5 |
| HCK |
| FCHO2 |
| FOXJ3 |
| ACADL |
| PTTG1IP |
| RP11-222K16.1 |
| ARHGAP30 |
| PAXIP1-AS2 |
| RP11-361L15.4 |
| GNAI2 |
| CTD-2651B20.5 |
| CXorf36 |
| TRIM69 |
| C10orf128 |
| TNFRSF9 |
| SNRK |
| TMEM246-AS1 |
| GALNT11 |
| BMP2K |
| 1-Mar |
| ZNF37CP |
| ENPP2 |
| PLXDC2 |
| THEMIS2 |
| SLC15A3 |
| RP11-89K18.1 |
| FKBP15 |
| PIK3R5 |
| CTD-2540F13.2 |
| ROBO4 |
| ZNF611 |
| KHK |
| CRYL1 |
| ARHGEF28 |
| SNX2 |
| RNF135 |
| PTER |
| ENPP7P8 |
| FAM114A2 |
| PLEKHO2 |
| AC011515.2 |
| SAMD9L |
| CDH5 |
| MS4A7 |
| GLYAT |
| HADHA |
| CCDC89 |
| RP11-798M19.6 |
| RP11-452C13.1 |
| C1orf54 |
| CX3CL1 |
| DGCR9 |
| RP11-232L2.2 |
| ZNF320 |
| MFAP3L |
| APOL3 |
| RP11-61L23.2 |
| SLC43A2 |
| AKAP2 |
| RAB42 |
| RSPH14 |
| TGOLN2 |
| MFSD1 |
| C14orf105 |
| CRYAB |
| DLEU7 |
| SLC2A9 |
| CXCR6 |
| PTAFR |
| TLR2 |
| RP13-644M16.1 |
| HHLA2 |
| LRRC19 |
| SNX3 |
| RNASET2 |
| TMEM38B |
| ITGAX |
| FASTKD3 |
| CANX |
| ABHD6 |
| RIT1 |
| SPP1 |
| RP5-1021I20.2 |
| LINC01550 |
| HIF1A-AS2 |
| LINC01094 |
| DENND1C |
| CREBL2 |
| RP11-155G14.5 |
| MSC-AS1 |
| UGT2B7 |
| KIAA1033 |
| TNFSF13 |
| HLA-A |
| PHYKPL |
| GGTA1P |
| MYCT1 |
| HECW2 |
| RP11-652L8.4 |
| USP2 |
| SLC23A3 |
| UGT3A1 |
| LILRB2 |
| BTN2A2 |
| CTD-2331H12.5 |
| RP11-489O18.1 |
| MYOM3 |
| INSR |
| GSAP |
| CYLD |
| RP11-556I14.1 |
| RP5-858L17.1 |
| GZMH |
| SMTNL2 |
| NFAM1 |
| GPR183 |
| TMEM133 |
| SLC31A2 |
| CLEC14A |
| SLITRK4 |
| ZNF189 |
| MYO9A |
| IFNAR2 |
| FLI1 |
| PPM1M |
| TMEM91 |
| QRFPR |
| AC006126.4 |
| IVNS1ABP |
| SLC17A4 |
| TLR8 |
| BNIP3 |
| DSCR3 |
| GBP4 |
| ADGRF5P2 |
| TRAV30 |
| EMCN |
| FEM1C |
| IMPA2 |
| HCST |
| VAMP5 |
| C10orf10 |
| CD48 |
| RP11-327J17.2 |
| ITGAV |
| HVCN1 |
| SLC25A30 |
| FAS |
| GRB10 |
| NNMT |
| PTP4A2P2 |
| ITGA1 |
| MIA2 |
| RGS14 |
| APOLD1 |
| MFAP3 |
| DNAJB9 |
| GPX3 |
| RP2 |
| CYB5A |
| SAMHD1 |
| RP11-598F7.5 |
| AC015977.6 |
| CSF1 |
| OGDH |
| BIVM-ERCC5 |
| CNEP1R1 |
| LINC01060 |
| TBC1D19 |
| HLA-DMB |
| APOBEC3G |
| UBTFL6 |
| TEK |
| GPR84 |
| C4orf47 |
| AC108463.1 |
| NHEJ1 |
| C1RL-AS1 |
| RP5-899E9.1 |
| CCL4 |
| GAREM |
| CD226 |
| SPAG4 |
| PDGFD |
| MEF2A |
| WDFY3-AS2 |
| CFLAR |
| FRZB |
| MAP3K7CL |
| MPP1 |
| AC108463.2 |
| PARK2 |
| GPD1 |
| AC015977.5 |
| CTD-3076M17.1 |
| AC010226.4 |
| EGLN1 |
| RP1-232P20.1 |
| VIM |
| ERAP1 |
| LDHA |
| AC002401.1 |
| CXCR2P1 |
| HOGA1 |
| MILR1 |
| ARL6IP5 |
| EPAS1 |
| RBM43 |
| FLT4 |
| CNN3 |
| BHMT |
| DARS-AS1 |
| KLF6 |
| CXCR4 |
| RP11-274H2.3 |
| SCARF1 |
| SIGLEC14 |
| GAL3ST4 |
| SLC11A1 |
| GGT8P |
| FHL5 |
| CX3CR1 |
| RALB |
| COTL1 |
| BDH2 |
| AOC1 |
| AC013275.2 |
| MPEG1 |
| LAIR1 |
| CTC-513N18.6 |
| TNFAIP8 |
| RP11-71E19.1 |
| TIE1 |
| RP11-6B4.1 |
| METTL9 |
| CPNE8 |
| MFNG |
| PKHD1 |
| OSTM1-AS1 |
| ORAOV1P1 |
| LINC00671 |
| MAOB |
| ATG4C |
| CLRN3 |
| HS1BP3 |
| F8 |
| SLC9A9 |
| CD300LF |
| AC004485.3 |
| RP11-8L8.2 |
| LINC01158 |
| KLRK1 |
| HLA-H |
| EXOC3L2 |
