# Supplementary material for: TIM-3 as a Prognostic Marker and a Potential Immunotherapy Target in Human Malignant Tumors: A Meta-Analysis and Bioinformatics Validation
Source: Front Oncol. 2021 Feb 22;11:579351. doi: 10.3389/fonc.2021.579351 (PMC7938756; doi:10.3389/fonc.2021.579351)
Supplement: Supplementary file 3 [file Table_2.docx]

Table S2. Characteristics of the included articles.

| First author | Country | Ethnicity | Age | Method | Study design | Stage | Histology | Cut-off values | N (E+%) | Follow-up time | CSS (HR with 95%CI) | OS (HR with 95%CI) | DFS (HR with 95%CI) | NOS |
| --- | --- | --- | --- | --- | --- | --- | --- | --- | --- | --- | --- | --- | --- | --- |
| Zhuang 2012 | China | Asian | 60 | IHC | Retrospective | 1-4 | Non-small-cell lung cancer | ≥25% | 30 (50%) | Average 34 months (1-78 months) |  | 4.481 (1.790-11.22) |  | 8 |
| Cao 2013 | China | Asian | 39 | IHC | Retrospective | 1-4 | Cervical cancer | 2-3 IRS scores | 43 (65.1%) | Median 45.2 months (5–60 months) |  | *P* < 0.05 (worse) |  | 6 |
| Piao 2013 | China | Asian | 57 | IHC | Retrospective | 1-4 | Prostate cancer | 3+ | 137 (20.4%) | Median 68 months (3-115 months) |  |  | 2.08 (1.07-4.16) | 8 |
| Yuan 2014 | China | Asian | 57 | IHC | Retrospective | 1-4 | Clear cell renal cell carcinoma | 3+-4+ | 137 (20.4%) | Mean 70 months (3-115 months) | 2.08 (1.07-4.16) |  |  | 8 |
| Jiang 2013 | China | Asian | 64 | IHC | Retrospective | 1-4 | Gastric cancer | HSCORE>0 | 305 (60%) | Median 40 months (3-135 months) |  | *P* = 0.010 (worse) |  | 9 |
| Yang 2015 | China | Asian | 65.32 | IHC | Retrospective | NA | Bladder urothelial carcinoma | HSCORE ≥100 | 100 (50%) | Median 44 months (3-60 months) |  | 5.514 (1.522–19.979) | 5.429 (1.484–19.858) | 9 |
| Zhou 2015 | China | Asian | 65 | IHC | Retrospective | 1-4 | Colorectal cancer | HSCORE≥200 | 201 (58.7%) | Median 61 months (2-120 months) |  | *P* < 0.0001 (worse) |  | 6 |
| Marcq 2016 | Belgium | European | NA | IHC | Retrospective | 1-4 | Malignant pleural mesothelioma | 1% in lymphoid aggregates | 36 (55.6%) | NA |  | 0.47 (0.29-0.76) |  | 7 |
| Ge 2017 | China | Asian | NA | ELISA | Retrospective | 2-3 | Osteosarcoma | Average serum soluble Tim-3 values | 120 (NA) | Median 37.1 months (7–80 months) |  | 2.12 (1.62–3.94) |  | 9 |
| Wu 2017 | China | Asian | 66 | IHC | Retrospective | NA | Metastatic prostate cancer | 6–8 scores | 139 (25.2%) | Median 22.1 months (NA-60 months) |  | 0.336 (0.133-0.846) |  | 8 |
| Hou 2017 | China | Asian | 58 | IHC | Retrospective | 2-3 | Esophageal squamous cell carcinoma | 3 points | 45 (22.2%) | < 100 months |  | 1.102 (0.292‑4.157) |  | 7 |
| Peng 2017 | China | Asian | 55.06 | IHC | Retrospective | 1-4 | Pancreatic cancer | 3-9 points | 50 (72%) | Median 10.3 months (NA-30 months) |  | 23.256 (5.435-100) |  | 7 |
| Li 2018 | China | Asian | 55.04 | ELISA | Retrospective | 1-4 | Hepatocellular carcinoma | >3000 pg/mL | 84 (60.7%) | Median 28 months (1–63 months) |  | 2.773 (1.474–5.219) |  | 8 |
| Su 2018 | China | Asian | 61 | IHC | Retrospective | 1-3 | Lung adenocarcinoma | 24% | 223 (48%) | Mean 76 months (4–101 months) |  | 2.04 (1.29–3.20) | 2.32 (1.44–3.73) | 9 |
| Wang 2018 | China | Asian | 61.6 | IHC | Retrospective | 1-4 | Gastric cancer | Median number of stained cells | 587 (49.9%) | Median 48 months (1-117 months) |  | 1.395 (1.078‑1.807) |  | 9 |
| Duan 2018 | China | Asian | 58 | IHC | Retrospective | 1-4 | Esophageal squamous cell carcinoma | 1% | 95 (37.9%) | Median 32 months (3–84 months) |  | 0.405 (0.148–1.103) |  | 9 |
| Burugu 2018 | Canada | European | NA | IHC | Retrospective | NA | Breast cancer | 1 | 3144 (10.5%) | Median 13 years | 0.64 (0.48–0.85) |  |  | 7 |
| Byun 2018 | Korea | Asian | 50 | IHC | Retrospective | 1-3 | Triple-negative breast cancer | 5% | 109 (84.4%) | Median 76 months (6–131 months) |  | 0.1129 (0.0323–0.3948) | 0.1072 (0.0319–0.3603) | 9 |
| Zhang 2019 | China | Asian | NA | IHC | Retrospective | NA | Renal cell carcinoma | 5% | 163 (56.4%) | Median 36.0 month (16.0–70.0 months) |  | 0.536 (0.245–1.172) |  | 8 |
| Chen 2019 | USA | European | 69 | IHC | Retrospective | 1-4 | Diffuse large B-cell lymphoma | 80 scores | 70 (32.9%) | Median 29 months (5–85 months) |  | 3.49 (1.40–6.15) |  | 7 |
| Fucikova 2019 | Czech Republic | European | NA | IF | Retrospective | NA | Ovarian cancer | NA | 50 (42%) | (0-120 months) |  | 1.41 (1.1–1.9) |  | 7 |
| Wang 2019 | China | Asian | 67 | IHC | Retrospective | 1-4 | Oral squamous cell carcinoma | 4 score | 36 (NA) | NA |  | 3.891 (2.786‐5.435) |  | 9 |
| Zhou 2019 | China | Asian | 45.8 | IHC | Retrospective | NA | Skull base chordoma | 278.2 cells/mm2 | 93 (NA) | Median 36.9 months ( 14–66 months) |  | 1.453 (0.921–1.607) | 2.353 (1.227-4.525) | 9 |
| Dancsok 2019 | Canada | European | 45 | IHC | Retrospective | NA | Nontranslocation-associated sarcoma | NA | 660 (NA) | NA-15 years |  | 0.992 (0.947-1.039) |  | 7 |
| Dancsok 2019 | Canada | European |  | IHC | Retrospective | NA | Translocation-associated sarcoma |  |  |  |  | 1.054 (0.840-1.323) |  |  |
| Babar 2019 | USA | European | 64.2 | RT-PCR | Retrospective | NA | Advanced esophageal adenocarcinoma | 3.9181 | 49 (NA) | Median 21.2 months (9.7-30.3 months) |  | 4.43 (1.70-11.53) |  | 8 |
| Wang 2019 | China | Asian | 59 | Multiplex Luminex assay | Prospective | 1-3 | Clear cell renal cell carcinoma | 5908 pg/ml | 182 (NA) | Median 66.1 months (1.1–134.1 months) |  | 3.12 (1.44–6.75) | 1.65 (0.61–4.40) | 8 |
| Hong 2019 | Korea | Asian | 64 | IHC | Retrospective | 1-4 | Oesophageal squamous cell carcinoma | 1% | 396 (50.8%) | Median 24.8 months (0.5–210 months) |  | 1.60 (1.13–2.27) | 1.52 (1.10–2.10) | 8 |

HR: hazard ratio; CI: confidence interval; CSS: cancer-specific survival; OS: overall survival; DFS: disease-free survival; IHC immunohistochemistry; ELISA: enzyme-linked immunosorbent assay; IF: immunofluorescence; RT-PCR: reverse transcriptase polymerase chain reaction; IRS: immunoreactivity-scoring; HSCORE system = stain intensity × the percentage of the stain cells; NA: not applicable; N: the number of the study population; E+: TIM-3 positive expression; NOS: New-Ottawa Scale.
